# High-risk characteristics of pathological stage I lung adenocarcinoma after resection: patients for whom adjuvant chemotherapy should be performed

**DOI:** 10.1016/j.heliyon.2023.e23207

**Published:** 2023-12-02

**Authors:** Lei-Lei Wu, Wen-Mei Jiang, Jia-Yi Qian, Jia-Yuan Tian, Zhi-Xin Li, Kun Li, Guo-Wei Ma, Dong Xie, Chang Chen

**Affiliations:** a Department of Thoracic Surgery, Shanghai Pulmonary Hospital, School of Medicine, Tongji University, 200092, PR China; b Sun Yat-sen University Cancer Center, State Key Laboratory of Oncology in South China, Collaborative Innovation Center for Cancer Medicine, Guangzhou, 510000, PR China

**Keywords:** Lung adenocarcinoma, Risk factors, Prognosis, adjuvant chemotherapy, Stage I

## Abstract

**Background:**

The objective of the present study was to identify patients with pathologic stage I lung adenocarcinoma (LUAD) who are at high risk of recurrence and assess the efficacy of adjuvant chemotherapy (ACT) in these individuals.

**Methods:**

A retrospective study was conducted on 1504 patients with pathologic stage I LUAD who underwent surgical resection at Shanghai Pulmonary Hospital and Sun Yat-sen University Cancer Center. Cox proportional hazard regression analyses were performed to identify indicators associated with a high risk of recurrence, while the Kaplan-Meier method and Log-rank test were employed to compare recurrence-free survival (RFS) and overall survival (OS) between patients with ACT and those without it.

**Results:**

Four independent indicators, including age (≥62 years), visceral pleural invasion (VPI), predominant pattern (micropapillary/solid), and lymphovascular invasion (LVI), were identified to be significantly related with RFS. Subsequently, patients were classified into high-risk and low-risk groups by LVI, VPI, and predominant pattern. The administration of ACT significantly increased both RFS (P < 0.001) and OS (P = 0.03) in the high-risk group (n = 250). Conversely, no significant difference was observed in either RFS (P = 0.45) or OS (P = 0.063) between ACT and non-ACT patients in the low-risk group (n = 1254).

**Conclusions:**

Postoperative patients with stage I LUAD with factors such as LVI, VPI, and micropapillary/solid predominant pattern may benefit from ACT.

## 1 Introduction

Lung cancer is one of the most common carcinomas and the leading cause of cancer-related mortality globally [1,2]. The 5-year overall survival (OS) rate for early-stage non-small cell lung cancer (NSCLC) might range from 73 % to 90 % when treated with surgical resection [[3], [4], [5]]. However, there remains a potential for tumor recurrence in patients with early-stage NSCLC after surgical resection, which accounts for the primary cause of cancer-related death. A multi-center research observed a relapsed rate of 26 % for stage I lung adenocarcinoma (LUAD) after complete surgical resection [6]. This phenomenon may be attributed to high-risk factors for tumor relapses after primary tumor resection and such patients need systematic treatment after the operation [7].

Several factors associated with poor survival in early-stage NSCLC patients have been proposed in the 8th edition of the American Joint Committee on Cancer (AJCC) Staging Manual [4,5]. Furthermore, adjuvant chemotherapy (ACT) has been demonstrated efficacy in reducing the death risk due to NSCLC recurrence. Nonetheless, not all patients undergoing surgery may derive benefits from ACT. Moreover, drug toxicity associated with ACT after surgery may increase the risk of non-cancer-related death in patients [8,9]. Currently, the significance of ACT in stage I NSCLC patients is unclear [10]. Previous reports propose that ACT should be considered for stage IB NSCLC with high-risk factors such as receiving wedge resection, spread through air spaces (STAS), poor differentiation, lymphovascular invasion (LVI), and visceral pleural invasion (VPI), but the potential benefit remains controversial [[11], [12], [13]]. LVI is defined as the formation of emboli in the lymphatic or vascular lumen by tumor cells. Several studies have suggested a correlation between LVI and an increased risk of lymph node metastasis or distant metastasis [14]. The concept of STAS was introduced as a new pattern of invasion in LUAD in 2015 and has been extensively studied, but its clinical utility in treatment decision-making remains controversial [15]. Besides, although numerous studies have identified these aforementioned factors as significant prognostic indicators for stage I NSCLC patients, the effect of adjuvant therapy has not been observed [[16], [17], [18], [19]].

Therefore, the present study aimed to explore the characteristics of stage I patients who are at high risk for recurrence. Additionally, we hypothesized that patients at high risk for relapses would benefit from ACT and therefore also investigated the role of ACT in this population.

## 2 Methods

### 2.1 Patient selection

The study was composed of patients from Sun Yat-sen University Cancer Center and Shanghai Pulmonary Hospital between 2015 and 2016. Patients were included in this study if they met all of the following criteria: (1) confirmed pathology of primary LUAD; (2) without metastasis to other organs or lymph nodes; (3) one primary tumor only; (4) the invasive component size of the tumor was smaller than 4 cm. Patients who fulfilled any of the following criteria were excluded from this study: (1) presence of adenocarcinoma in situ or minimally invasive adenocarcinoma; (2) invasion into parietal pleura, vessels, or ribs; (3) age <18 or age >80 years; (4) received neoadjuvant therapy; (5) perioperative mortality within one month after surgical resection; (6) the information of postoperative treatment was unknown. A total of 1504 patients were collected in this study. In addition, the TNM stage of those patients was identified according to the 8th Edition TNM classification system. The information on patients derived from clinical reports covered several parts: gender, age at surgery, smoking history, the extent of surgery, invasive component size, epidermal growth factor receptor (EGFR) mutation, the predominant pattern of tumor, STAS, VPI, LVI, and ACT.

### 2.2 Treatments

All patients included in the study underwent complete surgical resection, including lobectomy, sublobectomy (segmentectomy and wedge resection), and pneumonectomy with systemic lymphadenectomy. Patients without ACT treatment were classified as the non-ACT group, while those who received ACT were classified as the ACT group.

### 2.3 Follow-up and survival outcome

All patients in this study were followed up after operation, and relevant information was obtained by telephone interviews or medical records. The follow-up period ranged from 3.0 to 90.0 months, with an average of 59.6 months. Recurrence-free survival (RFS) was defined as the time from surgery to the first relapse or death. Recurrence was confirmed by tissue biopsy or detailed examinations including brain magnetic Resonance Imaging, chest computed tomography (CT), bone scan, or positron emission tomography-computed tomography (PET-CT). OS was defined as the time from surgery to death or last follow-up.

### 2.4 Variable declaration

According to the classification introduced by the International Association for the Study of Lung Cancer (IASLC), European Respiratory Society (ERS), and American Thoracic Society (ATS), histologic patterns of adenocarcinoma could be majorly classified as acinar, lepidic, papillary, micropapillary, and solid. The predominant histologic pattern was the one with the highest percentage of tumors. Tumors were then collapsed into three groups: lepidic predominant, acinar/papillary predominant, and micropapillary/solid predominant. In our study, invasive mucinous adenocarcinoma was also included, but adenocarcinoma in situ and minimally invasive adenocarcinoma were excluded. EGFR mutations were detected by liquid/tissue biopsy, mainly including a recurrent point mutation in exon 21 (L858R) and deletions in exon 19 (19-del).

### 2.5 Statistical analysis

The *t*-test and Mann-Whitney *U* test were conducted to compare continuous variables. The proportions of categorical outcomes were evaluated by Pearson's Chi-square test or Fisher's exact test. Univariable and multivariable Cox proportional hazard models were conducted to screen the independent prognostic factors. Predictors (*P* < 0.05) in the univariable analysis and known affecting-survival factors were brought into the multivariable analysis. Results of Cox regression were presented as hazard ratio (HR) and 95 % confidence interval (CI). In addition, the statistical significance was considered as a *P* < 0.05 on both sides. Interaction analysis was conducted to figure out the relationship between each prognostic factor. Patients' RFS and OS curves were drawn using the Kaplan-Meier method, and differences were compared by the log-rank test. Survival curves were constructed by R version 4.1.1 (https://www.r-project.org/) and statistical analyses were conducted by SPSS 25.0 (IMB-SPSS Inc, Armonk, NY).

## 3 Results

### 3.1 Patient characteristics

After selection, a total of 1504 resected LUAD patients were finally included in the present study (Shanghai Pulmonary Hospital, N = 1257; Sun Yat-sen University Cancer Center, N = 247), and the baseline information of patients was summarized in Table 1. The number of female patients was 857 (57.0 %). The median age at surgery of those patients was 61 years (interquartile range [IQR] 55–66). In the entire cohort, only 156 (10.4 %) underwent sublobectomy and 154 (98.7 %) of those patients had tumors smaller than or equal to 3 cm in size. Predominant patterns were as follows: lepidic 490 (32.6 %), acinar/papillary 866 (57.5 %), micropapillary/solid 99 (6.6 %), and mucinous 49 (3.3 %). The ACT group consisted of 427 patients (28.4 %), while the non-ACT group included 1077 patients (71.6 %). Significant differences in clinicopathologic factors were observed between these two groups. Patients treated with ACT were more likely to be younger, smokers, after sublobectomy, without EGFR mutations, with a micropapillary/solid predominant pattern, larger tumor size, STAS, VPI, and LVI.

**Table 1:** Clinicopathologic characteristics of the ACT and non-ACT groups.

Variables	Total (N = 1504)	ACT (N = 427)	Non-ACT (N = 1077)	*P* value
Gender				0.282
Male	647 (43.0 %)	193 (45.2 %)	454 (42.2 %)	
Female	857 (57.0 %)	234 (54.8 %)	623 (57.8 %)	
Age at surgery, years (IQR)	61 (55–66)	60 (54–64)	62 (55–67)	0.001
Smoking history				0.011
No	1246 (82.8 %)	337 (78.9 %)	909 (84.4 %)	
Yes	258 (17.2 %)	90 (21.1 %)	168 (15.6 %)	
Extent of surgery				＜0.001
Lobectomy	1346 (89.5 %)	407 (95.3 %)	939 (87.2 %)	
Sublobectomy	156 (10.4 %)	19 (4.5 %)	137 (12.7 %)	
Pneumonectomy	2 (0.1 %)	1 (0.2 %)	1 (0.1 %)	
Predominant pattern				＜0.001
Lepidic	490 (32.6 %)	89 (20.8 %)	401 (37.2 %)	
Acinar/Papillary	866 (57.5 %)	266 (62.3 %)	600 (55.8 %)	
Micropapillary/Solid	99 (6.6 %)	49 (11.5 %)	50 (4.6 %)	
Mucinous	49 (3.3 %)	23 (5.4 %)	26 (2.4 %)	
Invasive component size, cm (IQR)	2.0 (1.5–2.5)	2.5 (2.0–3.1)	1.8 (1.5–2.3)	＜0.001
VPI				＜0.001
Absent	1334 (88.7 %)	302 (70.7 %）	1032 (95.8 %)	
Present	170 (11.3 %)	125 (29.3 %）	45 (4.2 %)	
LVI				0.027
Absent	1488 (98.9 %)	418 (97.9 %)	1070 (99.4 %)	
Present	16 (1.1 %)	9 (2.1 %)	7 (0.6 %)	
STAS				0.013
Absent	1465 (97.4 %)	409 (95.8 %)	1056 (98.1 %)	
Present	39 (2.6 %)	18 (4.2 %）	21 (1.9 %)	
EGFR mutation				0.012
Negative	588 (39.2 %)	189 (44.3 %)	400 (37.1 %)	
19-del	399 (26.5 %）	117 (27.4 %)	282 (26.2 %)	
L858R	438 (29.1 %)	105 (24.6 %)	333 (30.9 %)	
Others	78 (5.2 %)	16 (3.7 %)	62 (5.8 %)	
Pathologic stage				＜0.001
IA	1213 (80.7 %)	222 (52.0 %)	991 (92.0 %)	
IB	291 (19.3 %)	205 (48.0 %)	86 (8.0 %)	

Data are expressed as n (%) or median (IQR). IQR, interquartile range; ACT, adjuvant chemotherapy; VPI, visceral pleural invasion; LVI, lymphovascular invasion; STAS, spread through air spaces. EGFR, epidermal growth factor receptor.

### 3.2 Univariable and multivariable analysis

To determine the independent prognostic indicators for the survival of patients after operation, we analyzed the RFS by using a Cox regression model. Multivariable analysis showed significant prognostic values for four factors for RFS: age ≥62 (HR 1.615, 95 % CI, 1.180–2.210, *P* = 0.003), predominant pattern (micropapillary/solid: HR 4.440, 95 % CI, 2.453–8.036, acinar/papillary: HR 2.413, 95 % CI, 1.525–3.816, all *P* < 0.001), VPI (HR 2.764, 95 % CI, 1.879–4.065, *P* < 0.001), LVI (HR 3.349, 95 % CI, 1.639–6.842, *P* = 0.001), and ACT (HR 0.614, 95 % CI, 0.416–0.904, *P* = 0.014) (Table 2). The effect of surgery on the RFS in our analysis is not statistically significant. Interaction analysis was performed to figure out the relationship between prognostic factors. We found that there were no interaction effects between VPI, LVI, and predominant pattern and age at surgery, indicating that these four factors were independent risk factors for patients’ prognosis. Besides, we found that adjuvant chemotherapy had interaction effects with VPI, LVI, and micropapillary/solid predominant pattern, respectively, and could improve the survival of patients with these factors (Supplementary Table 1).

**Table 2:** Univariable and multivariable analysis for RFS.

	Univariable analysis			Multivariable analysis		
Variables	HR	95%Cl	*P* value	HR	95%Cl	*P* value
Gender						
Male	1					
Female	0.739	0.545–1.001	0.051			
Age at surgery, years						
≤61	1			1		
≥62	1.707	1.254–2.325	0.001	1.615	1.180–2.210	0.003
Smoking history						
No	1					
Yes	1.187	0.807–1.745	0.384			
Extent of surgery						
Lobectomy	1					
Sublobectomy	0.966	0.584–1.596	0.892			
Pneumonectomy	5.624	0.787–40.209	0.085			
Predominant pattern						
Lepidic	1			1		
Acinar/Papillary	2.858	1.826–4.473	＜0.001	2.413	1.525–3.816	＜0.001
Micropapillary/Solid	6.557	3.758–11.439	＜0.001	4.440	2.453–8.036	＜0.001
Mucinous	1.131	0.339–3.766	0.841	1.248	0.372–4.185	0.719
Invasive component size, cm	1.519	1.260–1.831	＜0.001	1.219	0.977–1.521	0.080
VPI						
Absent	1			1		
Present	3.359	2.401–4.697	＜0.001	2.764	1.879–4.065	＜0.001
LVI						
Absent	1			1		
Present	6.263	3.197–12.268	＜0.001	3.349	1.639–6.842	0.001
STAS						
Absent	1					
Present	1.812	0.850–3.863	0.124			
EGFR mutation						
Negative	1					
19-del	0.934	0.641–1.361	0.722			
L858R	0.831	0.568–1.215	0.339			
Others	1.028	0.514–2.057	0.938			
ACT						
No	1			1		
Yes	1.160	0.837–1.607	0.372	0.614	0.416–0.904	0.014

VPI, visceral pleural invasion; LVI, lymphovascular invasion; STAS, spread through air spaces. EGFR, epidermal growth factor receptor; ACT, adjuvant chemotherapy.

### 3.3 Subgroup analyses

To further investigate the role of ACT in postoperative stage I LUAD patients, we stratified them into low-risk and high-risk groups based on pathologic characteristics that were independent prognostic factors for RFS. Patients with high-risk pathologic characteristics such as micropapillary/solid predominant pattern, LVI, and VPI were categorized into the high-risk group while others were placed in the low-risk group according to multivariable analysis. The characteristic information about patients in the low-risk and high-risk groups is shown in Table 3. There were 1254 patients in the low-risk group and 250 patients in the high-risk group. In the high-risk group, 218 (87.2 %) patients had one risk factor, 29 (11.6 %) patients had two risk factors, and 3 (1.2 %) patients had three risk factors.

**Table 3:** Clinicopathologic characteristics of the high-risk and low-risk groups.

	High-risk group			Low-risk group		
Variables	ACT (N = 159)	Non-ACT (N = 91)	*P* value	ACT (N = 268)	Non-ACT (N = 986)	*P* value
Gender			0.071			0.197
Male	72 (45.3 %)	52 (57.1 %)		121 (45.1 %)	402 (40.8 %)	
Female	87 (54.7 %)	39 (42.9 %)		147 (54.9 %)	584 (59.2 %)	
Age at surgery, years (IQR)	60 (54–65)	63 (57–68)	0.019	60 (54–64)	61 (55–67)	0.011
Smoking history			0.782			0.034
No	123 (77.4 %)	69 (75.8 %)		214 (79.9 %)	840 (85.2 %)	
Yes	36 (22.6 %)	22 (24.2 %)		54 (20.1 %)	146 (14.8 %)	
Extent of surgery			0.047			0.001
Lobectomy	154 (96.9 %)	82 (90.1 %)		253 (94.4 %)	857 (86.9 %)	
Sublobectomy	4 (2.5 %)	8 (8.8 %)		15 (5.6 %)	129 (13.1 %)	
Pneumonectomy	1 (0.6 %)	1 (1.1 %)		0 (0.0 %)	0 (0.0 %)	
Predominant pattern			0.001			＜0.001
Lepidic	13 (8.2 %)	2 (2.2 %)		76 (28.4 %)	399 (40.5 %)	
Acinar/Papillary	95 (59.7 %)	39 (42.9 %)		171 (63.8 %)	561 (56.9 %)	
Micropapillary/Solid	49 (30.8 %)	50 (54.9 %)		0 (0.0 %)	0 (0.0 %)	
Mucinous	2 (1.3 %)	0 (0.0 %)		21 (7.8 %)	26 (2.6 %)	
Invasive component size, cm (IQR)	2.5 (2.0–3.2)	2.5(2.0–2.9)	0.028	2.5 (2.0–3.0)	1.7 (1.5–2.2)	＜0.001
VPI			＜0.001			N/A
Absent	34 (21.4 %)	46 (50.5 %)		268 (100.0 %)	986 (100.0 %)	
Present	125 (78.6 %)	45 (49.5 %)		0 (0.0 %)	0 (0.0 %)	
LVI			0.528			N/A
Absent	150 (94.3 %)	84 (92.3 %)		268 (100.0 %)	986 (100.0 %)	
Present	9 (5.7 %)	7 (7.7 %)		0 (0.0 %)	0 (0.0 %)	
STAS			1.000			0.019
Absent	152 (95.6 %)	87 (95.6 %)		257 (95.9 %)	969 (98.3 %)	
Present	7 (4.4 %)	4 (4.4 %)		11 (4.1 %)	17 (1.7 %)	
EGFR mutation			0.606			0.166
Negative	76 (47.8 %)	46 (50.5 %)		113 (42.2 %)	354 (35.9 %)	
19-del	46 (28.9 %)	22 (24.2 %)		71 (26.5 %)	260 (26.4 %)	
L858R	32 (20.2 %)	22 (24.2 %)		73 (27.2 %)	311 (31.5 %)	
Others	5 (3.1 %)	1 (1.1 %)		11 (4.1 %)	61 (6.2 %)	

Data are expressed as n (%) or median (IQR). IQR, interquartile range; ACT, adjuvant chemotherapy; VPI, visceral pleural invasion; LVI, lymphovascular invasion; STAS, spread through air spaces. EGFR, epidermal growth factor receptor.

**Fig. 1:**
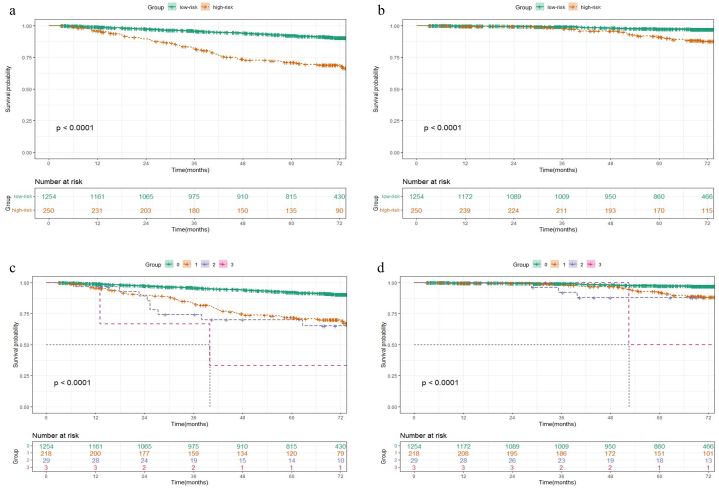
Kaplan–Meier survival curves for (a) RFS and (b) OS of patients between low-risk and high-risk groups, (c) RFS and (d) OS with different numbers of pathologic risk factors.

**Fig. 2:**
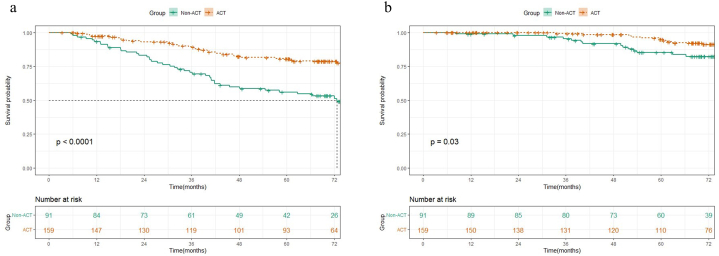
Kaplan–Meier survival curves for (a) RFS and (b) OS of patients in the high-risk group who received ACT and those who did not.

**Fig. 3:**
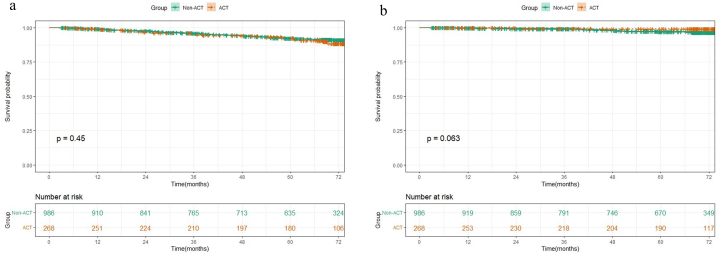
Kaplan–Meier survival curves for (a) RFS and (b) OS of patients in the low-risk group who received ACT and those who did not.

There were significant differences in both RFS and OS between low-risk and high-risk groups (Fig. 1a–b), and patients with more risk factors had a worse prognosis (*P* < 0.0001) (Fig. 1c–d). In the high-risk group, patients receiving ACT had significantly better RFS (5-year RFS, 80.8 % vs 56.0 %, HR 0.361, 95 % CI 0.225–0.579, *P* < 0.001) and OS (5-year OS, 94.2 % vs 85.3 %, HR 0.418, 95 % CI 0.186–0.942, *P* = 0.03) than those without ACT (Fig. 2a–b). In the low-risk group, no significant improvement in RFS (5-year RFS, 92.8 % vs 92.2 %, HR 1.192, 95 % CI 0.751–1.894, *P* = 0.45) or OS (5-year OS, 99 % vs 97 %, HR 0.280, 95 % CI 0.066–1.178, *P* = 0.063) was observed after receiving ACT (Fig. 3a–b).

## 4 Discussion

By analyzing the data collected from Sun Yat-sen University Cancer Center and Shanghai Pulmonary Hospital, we investigated the treatment strategies and survival of patients with stage I LUAD following surgery in the study. Our findings suggest that ACT may be beneficial for postoperative patients with risk factors for recurrence. Despite undergoing surgery, individuals with stage I LUAD still face the risk of recurrence, leading to diverse prognoses [19,20]. However, the evidence supporting postoperative ACT in patients with stage I NSCLC is not sufficient. Therefore, it is necessary to identify the high-risk group and explore whether this group can benefit from adjuvant therapy.

We identified five prognostic indicators, including VPI, age at surgery, LVI, the predominant pattern of LUAD, and ACT, which may influence postoperative RFS in patients with stage I LUAD based on the results of univariable and multivariable analyses. To further identify the population that could benefit from ACT, we found that the high-risk pathologic factors for recurrence including VPI, LVI, and micropapillary/solid predominant pattern could predict the prognostic value of ACT through interaction analysis and patients were divided into high-risk and low-risk groups according to these predictive factors. This stratification seems appropriate because VPI and LVI are well-known risk prognostic factors for early-stage NSCLC [4,14,21,22]. The micropapillary/solid predominant pattern is a significant prognostic factor for early-stage NSCLC, as previous studies have demonstrated that patients with this pattern are at higher risk of recurrence compared to those with other patterns [11,23]. While the age of the patient at the time of surgery is also an independent prognostic factor, patients receiving ACT after surgery are based primarily on risk factors in the postoperative pathology, not the age of the patients, therefore, we did not include it as a risk factor for grouping. Besides, older patients are often unable to tolerate ACT, which may influence the choice of ACT, and the interaction analysis showed that older patients could not benefit from chemotherapy compared to younger patients. Notably, the survival curves demonstrated that patients in the high-risk group could indeed derive benefits from adjuvant chemotherapy (ACT). On the contrary, long-term survival was relatively long in the low-risk group (5-year OS, >95 %), regardless of ACT.

Lobectomy has been established as the standard treatment for localized lung cancer since it was proposed in 1960 [24], but several recent studies concluded that sublobectomy could be an alternative to lobectomy with a comparable prognosis in stage I NSCLC [[25], [26], [27], [28]]. Consistent with these aforementioned findings, our study revealed that patients who underwent sublobectomy exhibited a similar prognosis to those who underwent lobectomy. Previous research has identified STAS as a risk factor influencing postoperative prognosis in stage I NSCLC patients [18,[29], [30], [31]], but no significant difference in prognosis was observed in this study. This may be due to the fact that the concept of STAS was only recently introduced in 2015 [15]. Moreover, STAS could be easily confused with artifacts such as loose tumor tissue fragments, which led to a bias toward the diagnosis [32,33]. Recently, X. Hou et al. proposed that resected pT2N0M0 NSCLC with wild-type EGFR has a worse prognosis and can derive survival benefits from ACT [13]. In our study, we incorporated the EGFR mutation and divided it into four groups: negative, 19-del, L858R, and others. The positive rate of EGFR mutations was about 60.8 %, and the most common types were 19-del and L858R. However, there were no significant differences in survival among the groups, which was consistent with the study of Y. T. Kim et al. [34].

In recent years, chest CT has replaced X-rays as a routine physical examination item, and more early-stage lung cancer can be detected and treated [35,36]. However, there is still significant heterogeneity in the prognosis of patients with the same stage of the disease and the use of ACT after surgery remains controversial. Current guidelines recommend routine adjuvant therapy for patients with stage IIB-IIIB NSCLC following surgery due to proven survival benefits [12,37]. For patients with stage pT2N0M0 (stage IB and IIA) NSCLC, adjuvant therapy is recommended only in patients with risk factors such as tumor size larger than 4 cm, VPI, vascular invasion, wedge resection, and poor differentiation [7]. However, some studies have shown that only patients with tumors larger than 4 cm in size can benefit from ACT [13]. The role of chemotherapy in stage I NSCLC is still unclear. Numerous studies have been conducted to investigate the treatment and prognosis of stage I NSCLC patients. However, most of these studies had limited sample sizes or failed to consider important factors such as gene mutations, receiving sublobectomy, or pathologic patterns [17,23,[38], [39], [40]]. In contrast, our study incorporated a large sample size and as many variables as possible. The results strongly supported that our definitions of low-risk and high-risk groups were appropriate and may assist in the decision-making process for ACT in stage I NSCLC.

It should be pointed out that our research still contained some limitations. First of all, despite the size of the sample involved in this study was large, the majority of patients were from Shanghai Pulmonary Hospital, which reduced the representativeness of the study. Moreover, as the study was retrospective, some bias was unavoidably included. Secondly, although the predominant patterns of tumors were recorded in the study, the exact proportion of each one was not described in the pathologic records. Thirdly, due to the large number of non-local patients, it is difficult to collect exact information about postoperative ACT, so the specific chemotherapy regimens for patients were not included in this study. Finally, due to the absence of pathologic biopsy in some patients, the diagnosis of recurrence mainly relied on the imaging examination and the judgment of multidisciplinary experts. Therefore, it is imperative to conduct multicenter and prospective studies to enroll more samples and variables to validate the potential benefit of ACT in stage I LUAD patients, and guide clinicians and patients in decision-making.

## 5 Conclusion

In summary, ACT may improve the prognosis in patients with pathologic stage I LUAD who have high-risk factors for recurrence such as LVI, VPI, or micropapillary/solid predominant pattern.

## Funding

This study was supported by Shanghai ShenKang Hospital Development Centre (SHDC22020218), National Key R&D Program of China (2019YFE0101200), 10.13039/501100001809National Natural Science Foundation of China (82272943), and the 10.13039/501100003399Science and Technology Commission of Shanghai Municipality (21Y11913400).

## Ethical statement

This study was reviewed and approved by the Ethics Committee of Shanghai Pulmonary Hospital (approval number: K22-209). Besides, considering the nature of the retrospective study, the ethics committee of Shanghai Pulmonary Hospital waived the patients' informed consent.

## Data availability statement

The data associated with our study has not been deposited into a publicly available repository. Data will be made available on request.

## CRediT authorship contribution statement

**Lei-Lei Wu:** Writing – original draft, Validation, Formal analysis, Data curation, Conceptualization. **Wen-Mei Jiang:** Writing – review & editing, Validation, Data curation. **Jia-Yi Qian:** Writing – original draft, Formal analysis, Data curation, Conceptualization. **Jia-Yuan Tian:** Writing – review & editing, Data curation. **Zhi-Xin Li:** Writing – review & editing, Data curation. **Kun Li:** Writing – review & editing, Validation, Data curation. **Guo-Wei Ma:** Supervision, Investigation. **Dong Xie:** Writing – review & editing, Writing – original draft, Supervision, Funding acquisition, Conceptualization. **Chang Chen:** Writing – review & editing, Supervision, Conceptualization.

## Declaration of competing interest

The authors declare that they have no known competing financial interests or personal relationships that could have appeared to influence the work reported in this paper.

## Abbreviations

NSCLC: Non-small cell lung cancer
LUAD: Lung adenocarcinoma
ACT: Adjuvant chemotherapy
HR: Hazard ratio
Cl: Confidence interval
OS: Overall survival
RFS: Recurrence-free survival
AJCC: American Joint Committee on Cancer
EGFR: epidermal growth factor receptor
VPI: Visceral pleural invasion
LVI: Lymphovascular invasion
STAS: spread through air spaces
IMA: Invasive mucinous adenocarcinoma
IQR: Interquartile range
CT: Computed tomography
PET-CT: Positron emission tomography-computed tomography
IASLC: International Association for the Study of Lung Cancer
ATS: American Thoracic Society
ERS: European Respiratory Society
