# Supplementary material for: High-risk characteristics of pathological stage I lung adenocarcinoma after resection: patients for whom adjuvant chemotherapy should be performed
Source: Heliyon. 2023 Dec 2;9(12):e23207. doi: 10.1016/j.heliyon.2023.e23207 (PMC10746451; doi:10.1016/j.heliyon.2023.e23207)
Supplement: Multimedia component 1 [file mmc1.docx]

**SUPPLEMENTARY TABLE 1** Interaction analysis of the prognostic factors for RFS

|  | **HR** | **95%CI** | ***P*-value** |
| --- | --- | --- | --- |
| Age at surgery, LVI | 0.722 | 0.187-2.793 | 0.637 |
| Age at surgery, VPI | 0.826 | 0.420-1.627 | 0.581 |
| Age at surgery, ACT | 1.287 | 0.665-2.490 | 0.455 |
| Age at surgery, Predominant pattern | 1.095 | 0.753-1.592 | 0.636 |
| LVI, VPI | 0.81 | 0.208-3.161 | 0.762 |
| LVI, ACT | 0.151 | 0.031-0.729 | 0.019 |
| LVI, Predominant pattern | 1.329 | 0.392-4.499 | 0.648 |
| VP, ACT | 0.406 | 0.230-0.716 | 0.002 |
| VPI, Predominant pattern | 0.673 | 0.395-1.149 | 0.147 |
| Predominant pattern |  |  |  |
| Lepidic | 1 |  |  |
| Acinar/Papillary | 1.428 | 0.979-2.083 | 0.064 |
| Micropapillary /Solid | 0.245 | 0.099-0.606 | 0.002 |
| Mucinous | 0.475 | 0.043-5.239 | 0.543 |

ACT, adjuvant chemotherapy; VPI, visceral pleural invasion; LVI, lymphovascular invasion; STAS, spread through air spaces. EGFR, epidermal growth factor receptor.
